# Synthesis of new water-soluble ionic liquids and their antibacterial profile against gram-positive and gram-negative bacteria

**DOI:** 10.1016/j.heliyon.2019.e02607

**Published:** 2019-10-10

**Authors:** Ali Niyazi Duman, Ismail Ozturk, Ayça Tunçel, Kasim Ocakoglu, Suleyman Gokhan Colak, Mine Hoşgör-Limoncu, Fatma Yurt

**Affiliations:** a Department of Material Science and Engineering, Ege University, Bornova, Izmir, 35100, Turkey; b Department of Pharmaceutical Microbiology, Faculty of Pharmacy, Izmir Katip Celebi University, Cigli, Izmir, 35620, Turkey; c Institute of Nuclear Science, Department of Nuclear Applications, Ege University, Bornova, Izmir, 35100, Turkey; d Department of Energy Systems Engineering, Faculty of Technology, Tarsus University, Tarsus, TR-33480, Turkey; e Faculty of Pharmacy, Department of Pharmaceutical Microbiology, Ege University, Bornova, Izmir, 35100, Turkey

**Keywords:** Inorganic chemistry, Organic chemistry, Bacteria, Microorganism, Biofilms, Antibacterial activity, Imidazolium salt, Ionic liquids, Imidazolium cation, Antibiofilm effect

## Abstract

A series of imidazolium bromide salts (NIM-Br 1a, 1b and 1c) bearing different lengths of alkyl chains were synthesized and their*in vitro* antibacterial activities were determined by measuring the minimum inhibitory concentration (MIC) values for *Staphylococcus aureus*, *Escherichia coli*, *Pseudomonas aeruginosa* and *Enterococcus faecalis*. In addition, these imidazolium derivatives were also evaluated against biofilm produced by these bacterial strains. All compounds were found to be effective against Gram-positive and Gram-negative bacteria, and also more effective on the *S. aureus* biofilm production than the others.

## 1 Introduction

Enhancing the resistance of pathogens against standard antimicrobial treatments causes to increase morbidity and mortality in a worldwide. Many important pathogens show resistant to clinically important classes of antibacterial agents. Therefore, designing new compounds such as Ionic liquids (ILs) which show antibacterial activities against *Staphylococcus aureus*, *Escherichia coli*, *Pseudomonas aeruginosa* and *Enterococcus faecalis*, are great importance. ILs are generally referred to be “green solvents” due to their low toxicity, low vapor pressure and remarkable chemical stability [1]. ILs have great range of cation–anion combinations which provide flexibility properties on their chemical structure [2]. They possess substituted nitrogen or phosphorus-containing cations (imidazole, pyridine, pyrrolidine, ammonium or phosphonium) and anions such as bromide (Br^−^), chloride (Cl^−^), hexafluorophosphate (PF_6_^-^), bis(trifluoromethane)sulfonimide (TFSI^−^) and tetrafluoroborate (BF_4_^-^). Polarization and ionization properties of these aromatic compounds enhance their pharmacokinetic features [3], and these properties improve their solubility and bioavailability. In this way, imidazole derivatives show pharmacological activities such as anticancer, antioxidant, anti-inflammatory, antiviral, antifungal, and antineoplastic well besides antimicrobial activities [3]. It was observed that some imidazole based drugs could harm the membrane surfaces of pathogenic microorganisms, especially when used at high concentrationsina short time. They can directlyinteract with double lipid layerinthe outer membranes ofthe microorganismsand increase their cell membrane permeability. This affects membrane structure of the bacterial cell, and reduces its resistance capacity by making it difficult to repair the membrane damage [4]. Moreover, these cationic compounds interrupt synthesis of microorganism's DNA or RNA, and causing the metal ions release thus inhibits activities of certain enzymes on the bacterial cells [5]. Recently, a strong relationship was found between the toxicity of the imidazolium based ILs and the alkyl-side chain length and cation ring planarity [6, 7, 8, 9, 10, 11]. Zheng et al. synthesized imidazolium type ionic liquid membranes, and investigated the effect of chemical structure, including carbon chain length of substitution and charge density of cations (mono- or bis-imidazolium) [12].

In some ILs, an anion induced toxicity, which was caused by the relationship between lipophilicity and the number of fluorine atoms, was observed. Their toxicities towards prokaryotic cells can be elucidated by this way [13, 14, 15, 16]. The increase in lipophilic character of ILs with increasing alkyl chain length could be explained by the fact that IL incorporation into biological membranes may cause disruption of membrane proteins (polar narcosis) [17].

Bacteria exist generally as not only free-floating planktonic organisms but also forming biofilms. A current definition of biofilm proposed by Rodney M. Donlan and J. William Costerton as follows; biofilm is a microbial derived sessile community characterized by cells that are irreversibly attached to a substratum or interface or to each other. They are embedded in a matrix of extracellular polymeric substances where they produce and exhibit an altered phenotype (compared to planktonic cells) with respect to growth rate and gene transcription [18]. This extracellular matrix can make slow drug-diffusion of biocides and antibiotics or can even act as a barrier due to its high viscosity. This formation is well developed as a communication system, which allows them to regulate microbial growth and metabolism. Biofilm formations are quite different from those of their planktonic forms. Eradication of biofilms on *E. coli, P. aeruginosa and S. aureus* was demonstrated by Ceri et al [19]. Compared to planktonic cells of the same organism, to eradicate biofilm formation requires 1000-fold higher concentrations of certain antibiotics must be used. It is found that biofilms play an important role on distribution of microbial diseases in the body. Eight percent of microbial infectious diseases, such as periodontitis, endocarditis and chronic cystic fibrosis lung disease, in humans caused by biofilms are well known [20]. On the other hand, biofilm-forming microorganisms have a tendency to develop by following themselves onto biotic or abiotic surfaces and thereafter onto surgical instruments, since exopolysaccharide glycocalyces provide a confluent protected biofilm [21]. Biofilm formation in infectious diseases causes serious problems in treatment, and imidazolium salts with their antimicrobial activities can have a role in preventing biofilm formation [22, 23, 24].

In this study, the antibacterial and antibiofilm activities of water-soluble imidazolium derivatives bearing different lengths of alkyl chains were evaluated. MIC values and antibiofilm properties of the synthesized compounds were determined against Gram-positive and Gram-negative bacterial strains.

## 2 Results and discussion

### 2.1 Lipophilicity of compounds

The lipophilicity of the new synthesized compounds was theoretically calculated by using ACD Chem Sketch Software. The obtained logP values of NIM-Br imidazolium derivatives (1a, 1c, 1b) are 7.80 ± 0.64, 8.56 ± 0.65, 9.92 ± 0.64, respectively. It is clearly seen that these values increase proportionally with increasing alkyl chain length. Lipophilicity value of the ITFSI compound was found to be 3.88 ± 0.9.

### 2.2 Antimicrobial activity

The MIC values (mean ± SD) determined for the substances and gentamicin on different bacteria are presented in Table 1. Control group MIC values of gentamicin are 0.12–1 μg/ml for *Staphylococcus aureus* ATCC 29213, 0.25–1 μg/ml for *Escherichia coli ATCC* 25922, 0.5–2 μg/ml for *Pseudomonas aeruginosa* ATCC 27853 and 4–16 μg/ml for *Enterococcus faecalis* ATCC 29212 according to the Clinical and Laboratory Standards Institute (CLSI). Our results showed that DMSO was inactive against bacteria at the used concentrations.

**Table 1:** Minimum inhibitory concentration for each of the compounds and Gentamicin.

Organism	ITFSI (μM)	NIM-Br (1a) (μg/ml)	NIM-Br (1b) (μg/ml)	NIM-Br(1c) (μg/ml)	GEN (μg/ml)
*Staphylococcus aureus* **ATCC 29213**	1.875 ± 0	8.33 ± 2.89	1.04 ± 0.36	4.17 ± 1.44	0.5 ± 0
*Escherichia coli* **ATCC 25922**	1.875 ± 0	260 ± 90.07	2.08 ± 0.72	8.33 ± 2.89	0.5 ± 0
*Pseudomonas aeruginosa* **ATCC 27853**	3.75 ± 0	312 ± 0	80 ± 0	53.3 ± 23,09	1 ± 0
*Enterococcus faecalis* **ATCC 29212**	3.75 ± 0	8.33 ± 2.89	1.25 ± 0	8.33 ± 2.89	8 ± 0

The results of the microdilution tests, which are shown in Table 1, indicatedthat NIM-Br imidazolium derivatives (1a, 1c, 1b) and imidazolium-TFSI salt (ITFSI) were exhibited antibacterial effects against Gram positive (*S. aureus* and *E. faecalis*) and Gram-negative (*E. coli* and *P. aeruginosa*) bacteria.

The compound 1b, which demonstrates the highest inhibitory effect against among these strains except *P. aeruginosa*, has the lowest MIC value. It also shows a good antimicrobial activity against *E. faecalis* when compared to commercial antimicrobial agent (Gentamicin). It was found that the compound demonstrates a higher antimicrobial activity on the Gram-positive strains than the Gram-negative strains. Also, *E. coli* bacteria was shown great interest, considering that Gram-negative strains are generally less responsive to antimicrobial agents due to their outer membrane, which behaves as an additional barrier on the bacteria cells.

Taking into account of these results, it may make an inference the antimicrobial activity of NIM-Br imidazolium derivatives (1a, 1c, 1b) depend on the length of the alkyl chain which is commonly known as “side chain effect” [25]. A strong broad spectrum of antimicrobial activity is observed when the compounds have alkyl chain lengths more than ten carbon atoms [26], and this is also supported by the MIC values obtained in our study. For instance, the compound 1b, which has a longer alkyl chain compared to others, shows a better antibacterial activity. Our results are quite similar to study reported by Carson et al.

The nature of the cell wall, the ligand, the coordination sites, the geometry of the compound, the positive charge density, hydrophilicity, lipophilicity, presence of co-ligand, pharmacokinetic factors also play a role in antimicrobial activity of compounds [27, 28]. These properties, as expected, may show antibacterial effects in different mechanisms. For example, when the compounds interact with the phosphate groups of the bacteria cell wall, the cationic charges on the imidazolium ring increase the antimicrobial activity due to the electrostatic attraction between the positively charged ligands and the negatively charged part on the cell wall [27, 28]. In other words, existence of the positive charge on the nitrogen atom in the imidazolium ring may have increased the affinity toward the microbial membrane surface of ILs [29]. In gram negative bacteria, the lipid membrane surrounding the bacteria cell and permitting passage of lipid soluble materials are known to be an important factor in controlling antimicrobial activity due to lipophilicity [30]. In this study, a low antimicrobial activity of the metal complexes is caused by their low lipophilicity, and this could be explained by decreased penetration of the complex through the lipid membrane.

### 2.3 Antibiofilm activity

The data regarding the antibiofilm activities of the compounds on planktonic form of bacterial cells and mature biofilm production are shown in Table 2. The results reveal that the compounds are more effective on *S. aureus* strain compared to the others. Imidazolium derivatives bearing alkyl chains have a wide broad-spectrum antimicrobial activity against bacteria in both the planktonic or biofilm structure. When the lengths of the substituent alkyl chains on the imidazolium derivatives increase, lipophilicity and thus the antimicrobial activities increase. The compounds bearing undecyl (C11) and hexadecyl (C16) chain lengths have higher antimicrobial activities, and their potential consistency amongst hydrophilicity and lipophilicity is vital for the antimicrobial activity of imidazolium salts. As shown in Table 2, antibiofilm activities at sub-MIC concentrations (MIC/2 and MIC/4) of each compound against bacterial strains were studied on planktonic cells and mature biofilm. It is considered that the antibiofilm activity of NIM-Br imidazolium derivatives (1a, 1c, 1b) depended on the alkyl chain length show consistency with MIC values obtained in our study.

**Table 2:** Antibiofilm effects of each compound against planktonic form of bacteria and mature biofilm.

		NIM-Br (1a)		NIM-Br (1b)		NIM-Br (1c)		ITFSI	
		MIC/2	MIC/4	MIC/2	MIC/4	MIC/2	MIC/4	MIC/2	MIC/4
*Staphylococcus aureus* **ATCC 29213**	Planktonic	↓	-	↓	↓	↓	↓	↓	↓
	Mature	↓	↓	↓	-	↓	-	↓	-
*Escherichia coli* **ATCC 25922**	Planktonic	-	-	-	-	-	-	-	-
	Mature	↓	-	↓	-	-	-	-	-
*Pseudomonas aeruginosa* **ATCC 27853**	Planktonic	-	-	-	-	-	-	-	-
	Mature	-	-	-	-	-	-	↓	-
*Enterococcus faecalis* **ATCC 29212**	Planktonic	-	-	-	-	↓	-	↓	↓
	Mature	-	↓	-	-	↓	-	-	-

MIC: Minimum inhibitory concentration. ↓: Inhibition of biofilm. -: No activity.

**Fig. 1:**
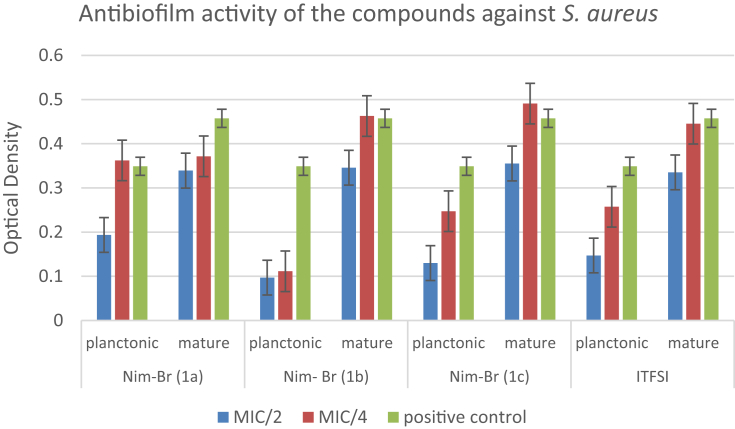
Antibiofilm activity of the compounds against *S. aureus*.

**Fig. 2:**
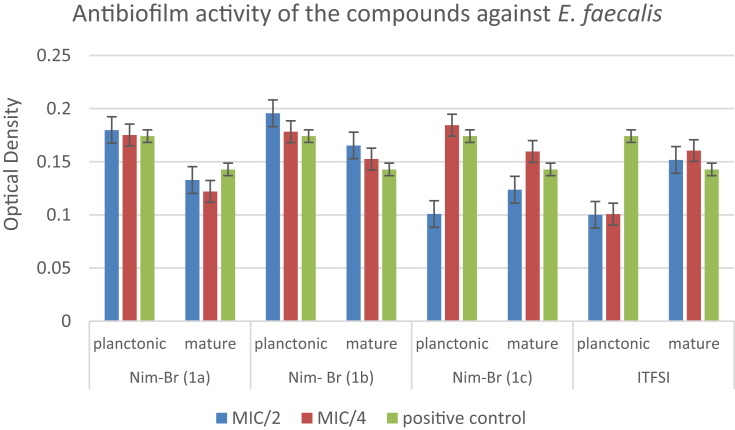
Antibiofilm activity of the compounds against *E. faecalis*.

**Fig. 3:**
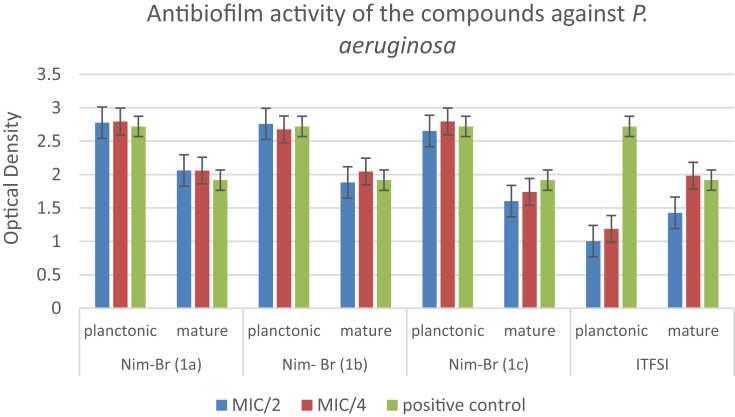
Antibiofilm activity of the compounds against *P. aeruginosa*.

**Fig. 4:**
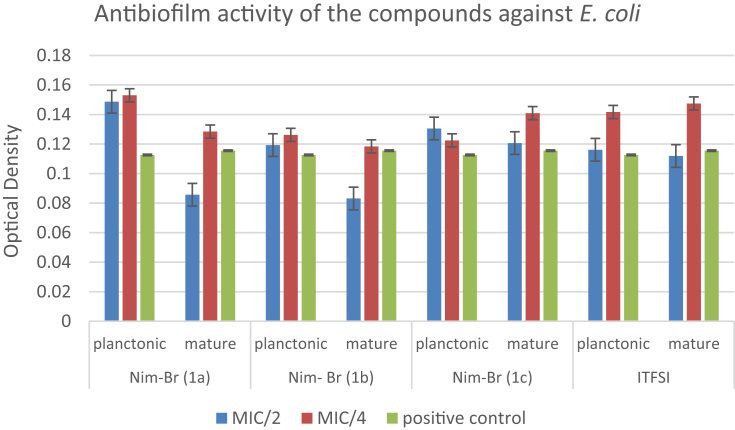
Antibiofilm activity of the compounds against *E. coli*.

In the study reported by Smith et al., no correlation was observed between the MICs and the efficacy of the antimicrobial agents toward microbial biofilms. Nevertheless, the antibiofilm potency was increased with increasing the alkyl chain length [31]. Except *E. coli*, the compounds exhibited good antibiofilm activity. Likewise, ITFSI exhibited good antibiofilm activity against all Gram-positive strains (*S. aureus* and *E. faecalis*) and Gram-negative *P. aeruginosa* (Figs. 1, 2, and 3). On the other hand, no biofilm formation was observed in *E. coli* (Fig. 4). The most toxic anion reported so far is bis (trifluoromethylsulfonyl) imide, and this situation is also explains the reason of our findings [32]. Antimicrobial activities were evaluated by determining the minimum inhibitory concentration (MIC) values for *E. coli* and *S. aureus*. The antibacterial activities of imidazolium type ionic liquids (ILs) and poly-ionic liquids (PILs) were improved in the presence of imidazolium cations which have higher charge density and long alkyl chains.

## 3 Conclusion

The results shown that antimicrobial efficiency of ionic liquids can be arranged by both altering alkyl chain length and modifying the head group. This may allow flexibility in the design of antimicrobial agents targeted at specific infections. As discussed before, increase in the toxicity of imidazolium-based salts is probably due to the higher lipophilicity, which can interact or disturb biological membranes. NIM-Br imidazolium derivatives that contain alkyl substituents with twelve and sixteen carbon length have shown that strong antimicrobial and antibiofilm activity against Gram-positive and Gram-negative bacteria. Antimicrobial and antibiofilm activities of ITFSI compound, which has methyl group on the cationic side, were also detected. The aim of this study was to assess the general appropriateness of imidazolium based ionic liquids as biofilm eradication agents for applications regarding antimicrobial activity.

Microbial biofilms present a great risk in clinical trials, and infectious diseases due to these biofilms are responsible for continuous financial loss at the industrial scale. In our study, we have demonstrated that these compounds have wide range antimicrobial and antibiofilm effects against bacterial strains, which can cause infectious diseases. This study showed that imidazolium derivatives are capable antimicrobial and antibiofilm agents, which can initiate approaches in novel clinical applications.

## 4 Materials and method

1,8-naphthalic anhydride, 1-(3-Aminopropyl) imidazole, 1-bromododecane, 1-bromohexadecane and 1-bromotetradecane used in the synthesis were purchased from Sigma-Aldrich, and they were used without further purification. ^1^H and ^13^C NMR spectra were recorded with a Bruker Avance III 400 MHz instrument. Thermogravimetric (TG) curves were recorded by a Shimadzu DTG-60H instrument in the temperature range of 25–1000 °C.

### 4.1 Synthesis of the imidazolium salts, (1a, 1b, 1c and ITFSI)

**Scheme 1:**
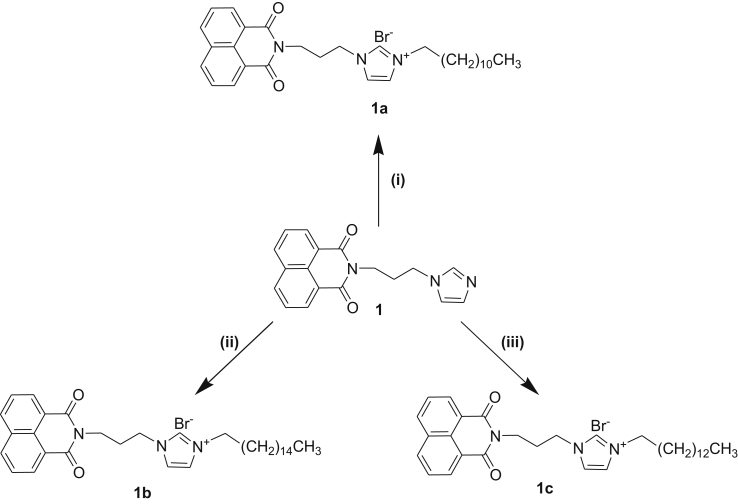
Synthesis of the imidazolium bromide salts, NIM-Br (1a, 1b, 1c). (i, ii, iii) CHCl_3_, inert atmosphere, reflux, overnight, 1-bromododecane (for 1a), 1-bromohexadecane (for 1b), 1-bromotetradecane (for 1c).

**Fig. 5:**
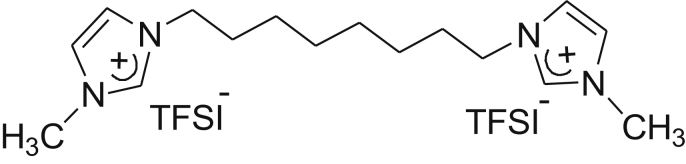
Molecular structure of octyl-bis(3-methylimidazolium) di(bis(trifluoromethane)sulfonamide) salt (ITFSI).

N-(3-propylimidazole)-1,8-naphthalene monoimide (1), imidazolium bromide salts (NIM-Br 1a, 1b and 1c) bearing different length of alkyl chains (Scheme 1) and octyl-bis(3-methylimidazolium)-di-(bis(trifluoromethane)sulfonamide) salt (ITFSI) (Fig. 5) were prepared according to previously reported procedures in the literature [33, 34, 35].

In a typical synthesis of an imidazolium bromide salt (1a, 1b, 1c), N-(3-propylimidazole)-1,8-naphthalene monoimide (1) was dissolved in CHCl_3_ under inert atmosphere, and then corresponding alkyl bromide was added dropwise to the solution. The reaction mixture was stirred and refluxed overnight. The mixture was allowed to cool down to room temperature. The obtained solid was filtered and was recrystallized from CH_2_Cl_2_/diethyl ether mixture. The compounds were characterized by ^1^H NMR, ^13^C NMR, FTIR and Thermogravimetric analysis (TGA) (see ESI for NMR, FTIR and TGA results, Figs. S1–S9).

### 4.2 Antibacterial activity

#### 4.2.1 Strains and growth media

*Staphylococcus aureus* ATCC 29213, *Escherichia coli* ATCC 25922, *Pseudomonas aeruginosa* ATCC 27853 and *Enterococcus faecalis* ATCC 29212 strains were studied. Bacteria were grown on Mueller–Hinton Agar (MHA) (Merck, Germany) at 35 °C for 24 h in the studies. All bacteria were stored in brain-heart infusion broth (Merck, Germany) with 10% glycerine at –80 °C.

#### 4.2.2 Determination of MIC values

Microdilution method was used to determine the MICs of active substances according to the Clinical and Laboratory Standards Institute (CLSI) criteria [36]. Gentamicin (I.E. Ulagay, Turkey) was used as the control antibiotic. Each of the experiments was made in triplicate. Bacterial strains were grown on MHA at 35 °C for 24h. A few colonies of bacteria were taken by sterile swabs and suspended with phosphate buffered saline (PBS) in sterile glass tubes. Bacterial suspensions in the tubes were adjusted to 0.5 McFarland turbidity with densitometer device (Den-1, Biosan, Latvia). Bacterial suspensions were diluted at the rate of 1/100. 50 μL of cation adjusted Mueller-Hinton II broth (Merck, Germany), and they were distributed in the wells of the sterile microplates. 50μL of the substances were added to the first wells, and 1/2 serial dilutions of the substances were performed. Bacterial suspensions (50 μL) were added to the wells, the microplates were incubated at 35 °C for 16–20 h. Media contamination, and growth controls were made on the same microplate. DMSO solution (50%, v/v) was used as co-solvent for the compounds and blank DMSO solution was studied as control group against bacteria in determining the antibacterial activity. After the incubation period, the minimum concentrations of active substances that inhibit bacterial growth visibly were determined as MICs. Mean MICs ± standard deviation rates (SD) were calculated.

#### 4.2.3 Biofilm assay

Antibiofilm effects of the active substances were determined by spectrophotometric microplate method as previously described [37] with minor modifications using bacterial suspensions (1.5 × 10^8^/mL), TSB (including 2.5% glucose), PBS and ethanol at different volumes. Antibiofilm activities were against planktonic form of bacteria and mature biofilm produced by bacteria were investigated. Bacterial strains were grown on MHA at 35 °C for 24 h. A few colonies of each strain were suspended in PBS and the suspensions were adjusted to 0.5 McFarland turbidity with densitometer. Tryptic soy broth (160 μL) (Merck, Germany) with 2.5% glucose was added to the microplate wells. DMSO (10%, v/v) was also investigated as co-solvent against bacteria in determining antibiofilm activity. Then the media were removed, and the wells were washed with 200 μL PBS three times. Following, the microplates were dried in ambient air. The wells were filled with 200 μL of methanol and they were left to stand still for 15 min. Then methanol was removed from the wells and the microplate was dried. Crystal violet solution (200 μL of 0.1%) was added into the wells and after 5 min the wells were washed with 200 μL tap water for three times and the microplates were dried. The wells were filled with 200 μL of absolute ethanol and incubated for 15 min. Then, spectrophotometric measurements were performed at 570 nm using Varioskan device (Thermo-Scientific, Germany). Antibiofilm effects of the substances on mature biofilm were determined after incubating the microorganisms without active substances for 24 h. Bacterial strains were grown on MHA at 35 °C for 24 h. Fresh colonies of each strain were suspended in PBS and the suspensions were adjusted to 0.5 McFarland turbidity with densitometer. Tryptic Soy Broth (TSB) (160 μL) including 2.5% glucose and 20 μL of bacterial suspensions were added to the wells of 96-well microplates. After 24 h of incubation, 20 μL of active substances were added into the wells that contain mature biofilm of the bacteria and the plates were incubated for 24 h at 35 °C. The media were removed, and the wells were washed with 200 μL PBS three times. The microplates were air-dried, and the wells were filled with 200 μL of methanol for 15 min. Then methanol was removed, and the microplates were dried. Crystal violet solution (200 μL, 0.1%) was added into the wells. After 5 min, the wells were washed with 200-μL tap water for, three times and the microplates were dried. The wells were filled with 200 μL of absolute ethanol and incubated for 15 min. Spectrophotometric measurements were performed at 570 nm using Varioskan device.

## Declarations

### Author contribution statement

Ali Niyazi Duman, Suleyman Gokhan Colak: Performed the experiments; Analyzed and interpreted the data.

Ayça Tunçel, İsmail Özturk: Performed the experiments; Analyzed and interpreted the data; Wrote the paper.

Mine Hoşgör-Limoncu: Analyzed and interpreted the data.

Fatma Yurt: Contributed reagents, materials, analysis tools or data; Wrote the paper.

Kasim Ocakoglu: Conceived and designed the experiments; Analyzed and interpreted the data; Contributed reagents, materials, analysis tools or data; Wrote the paper.

### Funding statement

This work was supported by the Ege University, Scientific Research Project (BAP), and Project Number: 16FBE008.

### Competing interest statement

The authors declare no conflict of interest.

### Additional information

No additional information is available for this paper.

## Appendix A Supplementary data

Supplementary Info_23-09-2019 Supplementary Info_23-09-2019
