# Supplementary material for: Synthesis of new water-soluble ionic liquids and their antibacterial profile against gram-positive and gram-negative bacteria
Source: Heliyon. 2019 Oct 10;5(10):e02607. doi: 10.1016/j.heliyon.2019.e02607 (PMC6812458; doi:10.1016/j.heliyon.2019.e02607)
Supplement: Supplementary Info_23-09-2019 [file mmc1.docx]

**SUPPLEMENTARY INFORMATION**

**Synthesis of New Water-Soluble Ionic Liquids and their Antibacterial Profile against Gram-Positive and Gram-Negative Bacteria**

Ali Niyazi Duman^1^, Ismail Öztürk^2^, Ayça Tunçel^3^, Kasim Ocakoglu^4*^, Mine Hoşgör-Limoncu^5^, Fatma Yurt^1,3*^

^1^Department of Material Science and Engineering, Ege University, Bornova, 35100, Izmir, Turkey.

^2^Department of Pharmaceutical Microbiology, Faculty of Pharmacy, Izmir Katip Celebi University, 35620, Cigli, Izmir, Turkey.

^3^Institute of Nuclear Science, Department of Nuclear Applications, Ege University, Bornova, 35100, Izmir, Turkey.

^4^Department of Energy Systems Engineering, Faculty of Technology, Tarsus University, TR-33480, Tarsus, Turkey.

^5^Faculty of Pharmacy, Department of Pharmaceutical Microbiology, Ege University, Bornova, 35100, Izmir, Turkey.

*Corresponding authors: [fatma.yurt.lambrecht@ege.edu.tr](mailto:fatma.yurt.lambrecht@ege.edu.tr) (F. Yurt)

kasim.ocakoglu@tarsus.edu.tr (K. Ocakoglu)


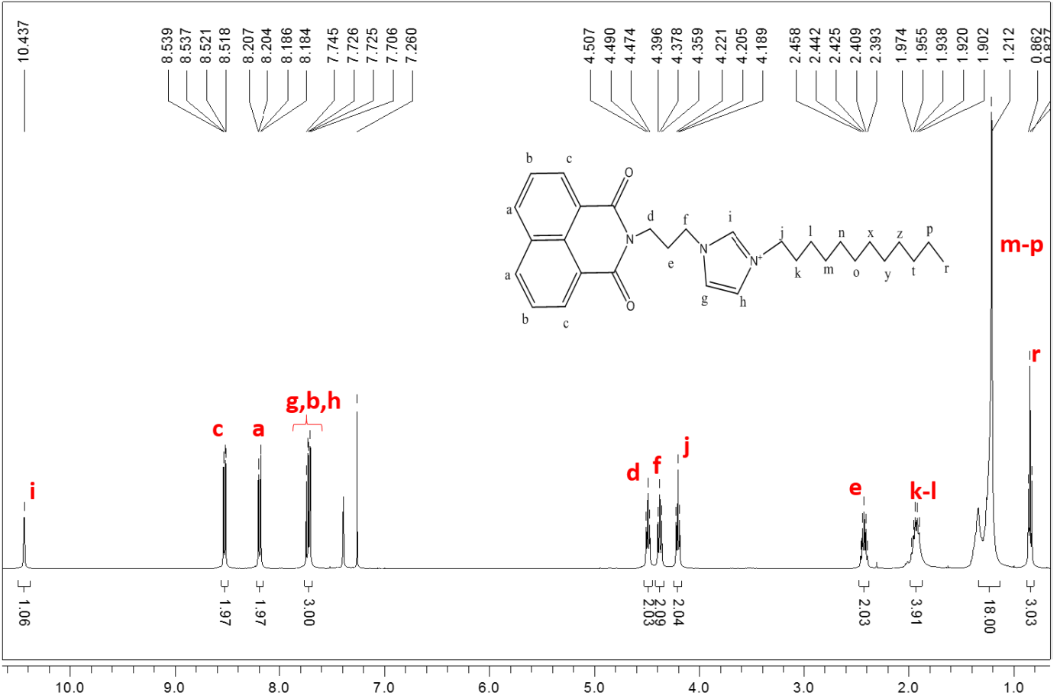


**Figure S1.** ^1^H NMR spectrum of **NIM Br 1a** in CDCl_3_.

**Figure S2.** ^13^C-NMR spectrum of **NIM Br 1a** in CDCl_3_.

**NMR results of the compound NIM Br 1a**

^1^H NMR (δ_H_, ppm, 400 MHz, CDCl_3_): 10.48 (s, 1H, Ar); 8.53 (dd, 2H, *J*=1.04 Hz, 7.28 Hz, Ar); 8.20 (dd, 2H, *J*=1.04 Hz, 7.28 Hz, Ar); 7.74-7.70 (m, 3H, Ar); 4.49 (t, 2H, *J*= 6.6 Hz); 4.38 (t, 2H,*J*= 7.46 Hz); 4.20 (t, 2H, *J*=6.5 Hz); 2.42 (q, 2H, *J*= 13.2 Hz); 1.97-1.89 (m, 4H); 1.21 (m, 17H,); 0.84 (t, 3H, *J*= 6.9 Hz). ^13^C NMR (δ_c_, ppm, 400 MHz, CDCl_3_): 164.3, 137.7, 134.3, 131.5, 128.1, 127.0, 122.3, 122.1, 121.6, 50.3, 47.9, 36.8, 31.8, 30.2, 29.5, 29.3, 29.0, 26.2, 26.1, 22.6, 14.0.


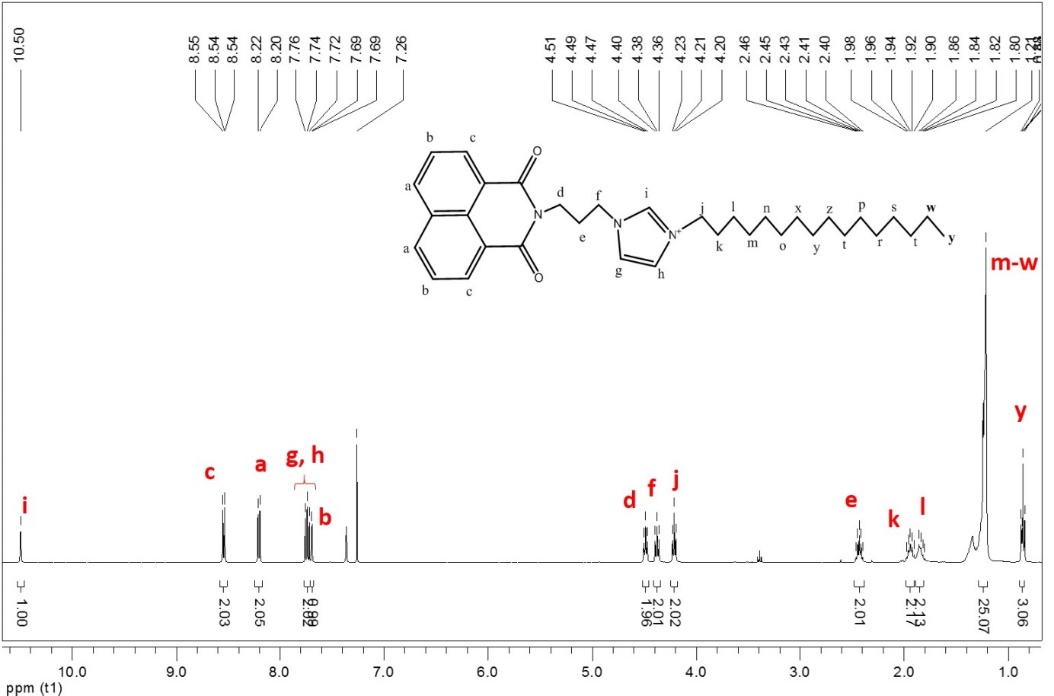


**Figure S3.** ^1^H NMR spectrum of **NIM Br 1b** in CDCl_3_.

**Figure S3.** ^13^C-NMR spectrum of **NIM Br 1b** in CDCl_3_.

**NMR results of the compound NIM Br 1b**

^1^H NMR (δ_H_, ppm, 400 MHz, CDCl_3_): 10.49 (s, 1H, Ar); 8.53 (dd, 2H, *J*=1.04 Hz, 7.28 Hz, Ar); 8.20 (dd, 2H, *J*=1.04 Hz, 7.28 Hz, Ar); 7.75-7.71 (t, 2H,*J*=8.2 Hz, Ar); 7.69 (t, 1H, *J*=0.8 Hz, Ar); 4.48 (t, 2H, *J*= 6.6 Hz); 4.38 (t, 2H,*J*= 7.46 Hz); 4.20 (t, 2H, *J*=6.5 Hz); 2.43 (q, 2H, *J*= 13.2 Hz); 1.97-1.90 (m, 2H); 1.85-1.80 (m, 2H); 1.21 (m, 25H,); 0.84 (t, 3H, *J*= 6.9 Hz). ^13^C NMR (δ_c_, ppm, 400 MHz, CDCl_3_): 164.3, 137.7, 134.4, 131.5, 128.1, 127.0, 122.2, 122.1, 121.5, 50.3, 47.9, 36.8, 31.9, 30.2, 29.67, 29.64, 29.61, 29.5, 29.39, 29.34, 29.03, 29.01, 26.3, 22.6, 14.0.


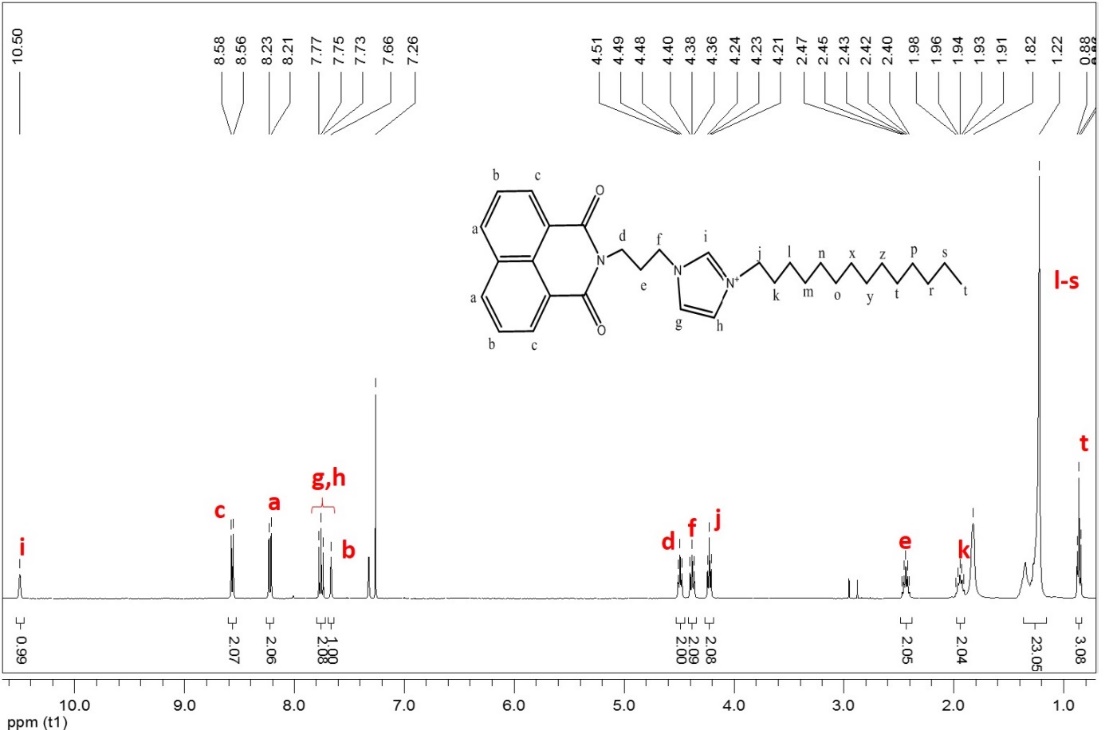


**Figure S5.** ^1^H NMR spectrum of **NIM Br 1c** in CDCl_3_.

**Figure S6.** ^13^C-NMR spectrum of **NIM Br 1c** in CDCl_3_.

**NMR results of the compound NIM Br 1c**

^1^H NMR (δ_H_, ppm, 400 MHz, CDCl_3_): 10.49 (s, 1H, Ar); 8.57-8.55 (d, 2H, *J*=3.6 Hz, Ar); 8.23-8.21 (d, 2H, *J*=4 Hz, Ar); 7.77-7.73 (t, 2H, *J*=8 Ar); 7.66 (s, 1H, Ar); 4.49 (t, 2H, *J*= 8 Hz); 4.38 (t, 2H,*J*= 8.4 Hz); 4.22 (t, 2H, *J*=6.5 Hz); 2.43 (q, 2H, *J*= 13.2 Hz); 1.97-1.90 (m, 2H); 1.22 (m, 23H,); 0.86 (t, 3H, *J*= 6.9 Hz). ^13^C NMR (δ_c_, ppm, 400 MHz, CDCl_3_): 164.4, 137.9, 134.4, 131.6, 128.1, 127.0, 122.2, 121.4, 50.4, 47.9, 31.9, 30.2, 29.66, 29.63, 29.60, 29.5, 29.39, 29.33, 29.03, 28.9, 26.3, 22.6, 14.0.


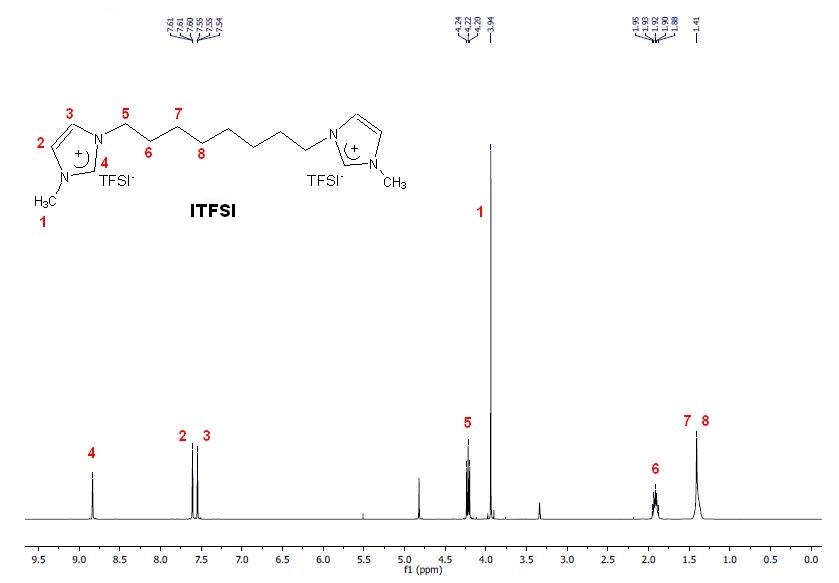


**Figure S7.** ^1^H NMR spectrum of **ITFSI** in CH_3_OD.

**NMR results of the compound ITFSI**

^1^H NMR (CH_3_OD) δ ppm: 8.83 (s, 2H, NC*H*N), 7.61 (t, *J* = 1.6 Hz, 2H, NC*H*CHN), 7.55 (t, *J* = 2 Hz, 2H, NCHC*H*N), 4.22 (t, *J* = 7.6 Hz, 4H, NC*H2*CH2), 3.94 (s, 6H, –NC*H3*), 1.97-1.84 (m, 4H, NCH2C*H2*), 1.41 (brs, 8H, -C*H2*C*H2*C*H2*C*H2*-).^13^C NMR (CD_3_OD) δ ppm: 136.42 (N*C*HN), 124.73 (TFSI), 123.67 (N*C*HCHN), 122.91 (NCH*C*HN), 121.57 (TFSI), 118.36 (TFSI), 115.18 (TFSI), 49.32 (N*C*H_2_CH_2_), 35.27 (–N*C*H_3_), 29.52 (–*C*H_2_*C*H_2_–), 28.33 (NCH_2_*C*H_2_–), 25.63 (NCH_2_CH_2_*C*H_2_).

NIM-Br 1a

NIM-Br 1b

NIM-Br 1c

**Figure S8.** FTIR spectra of imidazolium bromide salts (NIM Br 1a, 1b and 1c).

**NIM Br 1a.** Imidazole rings; symmetrical stretching vibration (3483 cm^-1,^ ν^s^ C-H_2_) and asymmetrical stretching vibration of C-H aromatic bonds (3412.3 cm^-1^, ν^as^ C-H_2_), symmetrical alkyl stretching vibration (2850 cm^-1,^ ν^s^ CH_2_) and asymmetrical alkyl stretching vibration (2920.8 cm^-1^, ν^as^ CH_2_) of C-H aliphatic bonds in the methylene (-CH_2_-), C=O stretching vibration (1658.9 cm^-1^ ν C=O), C=N stretching vibration / aromatic amines (1340.4-1163.5 cm^-1^,νC-N), C-N stretching vibration / aliphatic amines (1163.5-1037.7 cm^-1^,νC-NH_2_), C–H stretching bonds seen only in long chain alkanes, (778.27 cm^-1^) stretching vibration of Br-C bond (636.74- 542.39 cm^-1^).

**NIM Br 1b.** Obvious peaks such as imidazole rings; symmetrical stretching vibration (3475.2 cm^-1,^ ν^s^ C-H_2_) and asymmetrical stretching vibration of C-H aromatic bonds (3412.3 cm^-1^, ν^as^ C-H_2_), symmetrical alkyl stretching vibration (2854 cm^-1,^ ν^s^ CH_2_) and asymmetrical alkyl stretching vibration (2920.8 cm^-1^, ν^as^ CH_2_) of C-H aliphatic bonds in the methylene (-CH_2_-), C=O stretching vibration (1662 cm^-1^ ν C=O), C=N stretching vibration / aromatic amines (1344.4-1163.5 cm^-1^,νC-N), C-N stretching vibration / aliphatic amines (1163.5-1033.8 cm^-1^,νC-NH_2_), C–H stretching bonds seen only in long chain alkanes, (778.27 cm^-1^) stretching vibration of Br-C bond (626.74- 542.39 cm^-1^).

**NIM Br 1c.** Imidazole rings; symmetrical stretching vibration (3480.2 cm^-1,^ ν^s^ C-H_2_) and asymmetrical stretching vibration of C-H aromatic bonds (3409 cm^-1^, ν^as^ C-H_2_), symmetrical alkyl stretching vibration (2851.6 cm^-1,^ ν^s^ CH_2_) and asymmetrical alkyl stretching vibration (2922.7 cm^-1^, ν^as^ CH_2_) of C-H aliphatic bonds in the methylene (-CH_2_-), C=O stretching vibration (1661.7 cm^-1^ ν C=O), C=N stretching vibration / aromatic amines (1341.5-1167.5 cm^-1^,νC-N), C-N stretching vibration / aliphatic amines (1167.5-1033.1 cm^-1^,νC-NH_2_), C–H stretching bonds seen only in long chain alkanes, (780.1 cm^-1^) stretching vibration of Br-C bond (637.78- 542.9 cm^-1^).


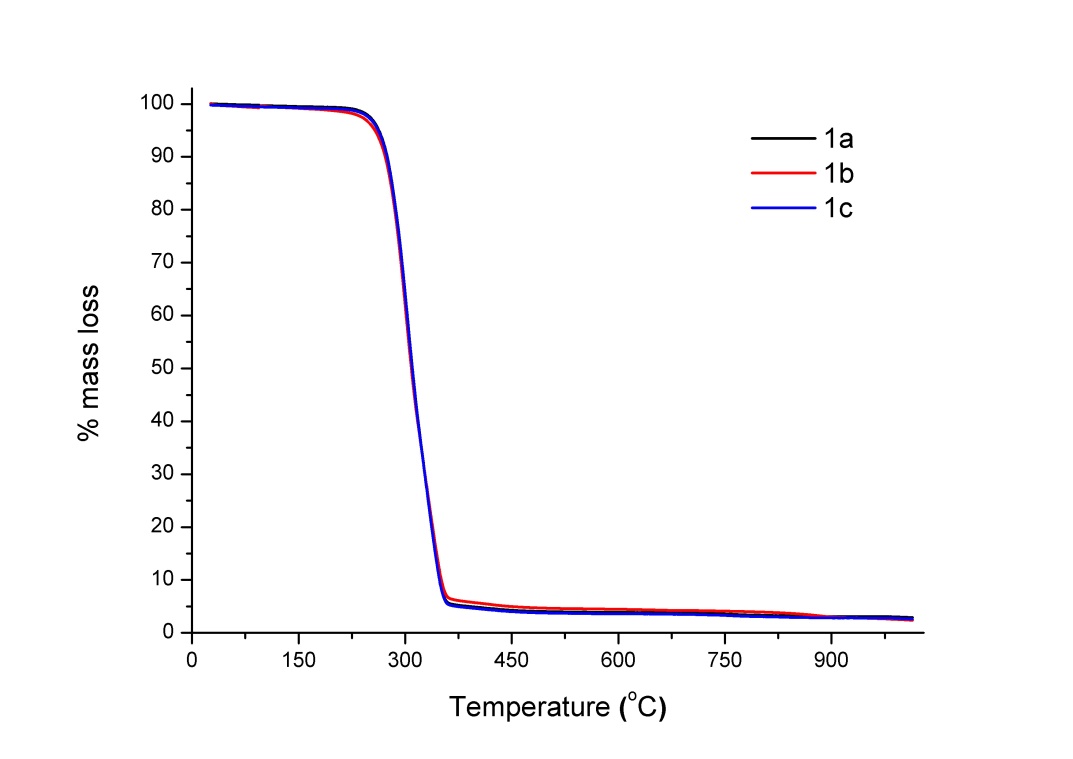


**Figure S9.** Characteristic decomposition curves of imidazolium bromide salts (NIM-Br 1a, 1b and 1c) determined by TGA measurements.
